# Utility of non-contrast Dual Energy Computed Tomography in diagnosis of differentiated thyroid cancer – two case study

**DOI:** 10.1186/s40644-023-00555-w

**Published:** 2023-04-18

**Authors:** Adam Daniel Durma, Marek Saracyn, Arkadiusz Zegadło, Grzegorz Kamiński

**Affiliations:** 1 Department of Endocrinology and Radioisotope Therapy, Military Institute of Medicine - National Research Institute, Warsaw, Poland; 2 Department of Medical Radiology, Military Institute of Medicine - National Research Institute, Warsaw, Poland

**Keywords:** DECT, Dual Energy Computed Tomography, Differentiated thyroid cancer, DTC

## Abstract

**Background:**

Dual Energy Computed Tomography (DECT) is a technology that allows for viewing computed tomography spectral images. This method, due to ability of presenting specific elements and substances (like water, calcium and iodine), can be used to locate selected type of tissues. Thyroid tissue due to being rich in endogenous iodine, can be located even without administration of contrast agent.

**Case presentation:**

In presented cases authors used a feature of accumulating endogenous iodine in thyroid derivative tissue for diagnosis of differentiated thyroid cancer metastases. In Patient One DECT was a decisive parameter qualifying for the surgery. Due to use of DECT in Patient Two it was possible to directly localize thyroid cancer metastases, which was unfeasible using standard techniques (scintigraphy and [^18^ F]FDG PET/CT). It helped to perform targeted biopsy and confirm diagnosis of thyroid cancer metastases, allowing to introduce treatment with sorafenibe.

**Conclusion:**

DECT confirmed its utility in locating thyroid tissues, including differentiated thyroid cancer (DTC) metastases. The method could be used in the future, especially in borderline or ambiguous cases with no localization of DTC in ultrasonography, RAI scintigraphy, or [^18^ F]FDG PET/CT, and among patients having contraindications for contrast-CT.

## Introduction

Dual energy computed tomography (DECT) is an advanced, modern, and unique technology for the qualitative and quantitative evaluation of materials with different atomic numbers (Z). It was used for the first time in diagnostic imaging in 2006 [1].

Comparing to standard computed tomography where materials with different elemental compositions are represented by identical pixel values on a CT image (depending on the mass density), in dual-energy CT an additional attenuation measurement is obtained with a second x-ray spectrum (with different “energy”), allowing to differentiate the materials [2]. The method principles are based on the interaction of an atom with a photon of ionizing radiation. The common elements found in human body, such as hydrogen (Z = 1), carbon (Z = 6), nitrogen (Z = 7) and oxygen (Z = 8) have a very similar atomic numbers and too few electrons on the electron shells to cause a photoelectric effect. The use of an iodine-based contrast agent (Z = 53) enables a photoelectric effect between the photon and the iodine atoms in the tissues [3]. There are some variants of DECT technique that can be performed, and one of them is Spectral Computed Tomography (SpCT). The clinical use of SpCT is based on different absorption of photon energy spectra (ranging from 80 keV to 140 keV), and calculations made by dedicated software. The detailed and thorough explanation of the DECT methods and variants is beyond the scope of this paper, although can be found in McCollough et al. publication [2]. There is also a number of publications showing other benefits of using SpCT in many areas of medicine: oncology, emergency, orthopaedics or vascular diagnostic, however those studies were mainly based on images acquired after intravenously administration of iodine contrast agent [4–7]. There were also some studies dedicated to assess thyroid tissue (thyroid nodules, thyroid cancer metastasis) [8–10]. Those studies were comparing SpCT to standard CT or USG [11–16], and the results showed possible utility of the method in diagnosis of thyroid diseases. However, the main disadvantage of those studies was using the iodine contrast agent, which is contraindicated in some group of patients, and can postpone differentiated thyroid cancer (DTC) radioiodine (RAI) therapy.

SpCT tests without iodine contrast agent are not typically performed, so there are limited number of data available. Therefore some researchers tried to estimate iodine concentration in human thyroid tissue [17], or predict extrathyroidal extension and recurrence of PTC [18]. Moreover Bunch et al., in a retrospective study including 20 dual energy parathyroid computed topographies assessed the attenuation of thyroid tissue, parathyroid lesions, and sternocleidomastoid muscle [19]. With use of 40 keV they discriminated parathyroid lesions from thyroid tissue by significantly increasing thyroid attenuation and associated contrast-to-noise, proving utility of the method in locating iodine-rich tissues, even with use of lower energy spectra. Obtained results confirmed that the method can be used to detect endogenous iodine of thyroid derived tissues. Therefore, we have made an attempt to use this technique to diagnose and control patients with differentiated thyroid cancer. Cases presented in the manuscript showed that this is an interesting method with a great potential in the field of endocrinology and diagnostic imaging.

## Methods and protocol

Tests were performed using a Discovery CT 750 HD kVp fast switching single source SpCT scanner (GE Healthcare, WI, USA). The studies were sent from the scanner to the commercially available Advantage Workstation server 4.7 (GE Healthcare) commercial workstation according to the GSI General protocol in GE Healthcare Workstation 4.7 software. The results (µg × 100 / cm3) were read automatically and displayed the mean concentrations of endogenous iodine in the ROI areas corresponding to the measurement sites. The tests were color-coded (Inverse Gray and GE Colormap) to assess position and increase contrast, option available in the software GSI General at the AW.

## Case 1

**Fig. 1:**
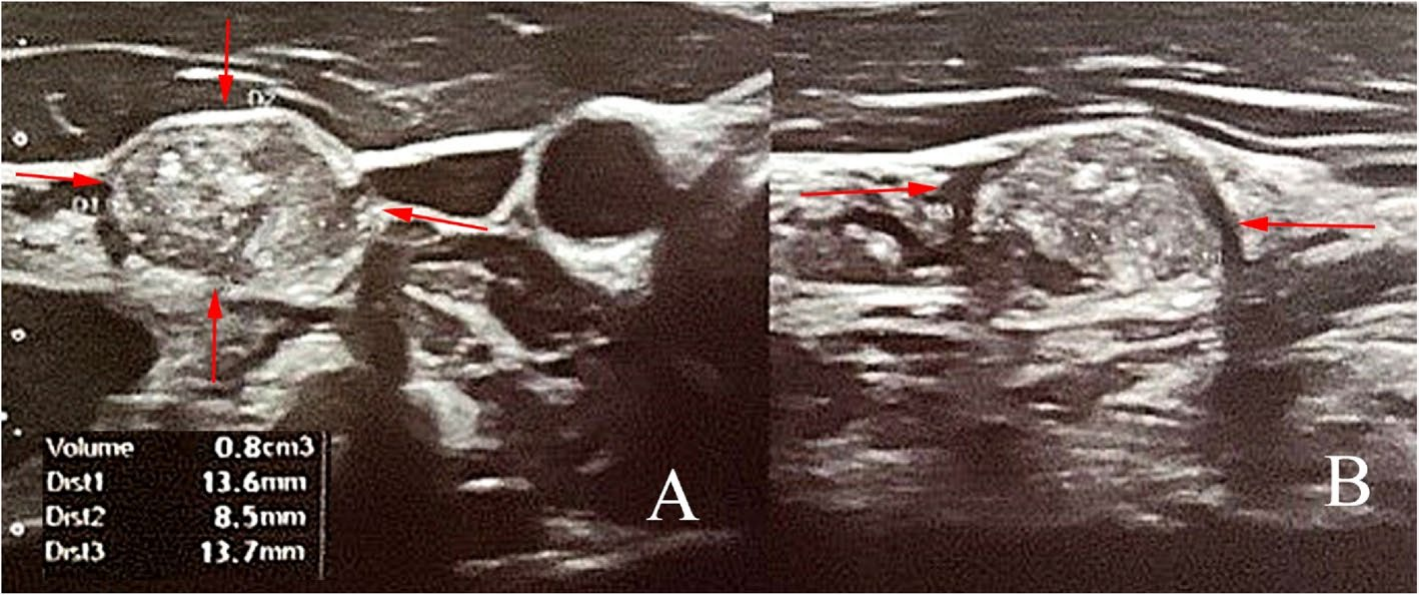
Ultrasound of the neck after thyroidectomy. Suspicious lymph node behind sternocleidomastoid muscle, with following features: solid, hyperechogenic, mixed vascularity type and microcalcifications. (**A**) Transverse section (**B**) Sagittal section

**Fig. 2:**
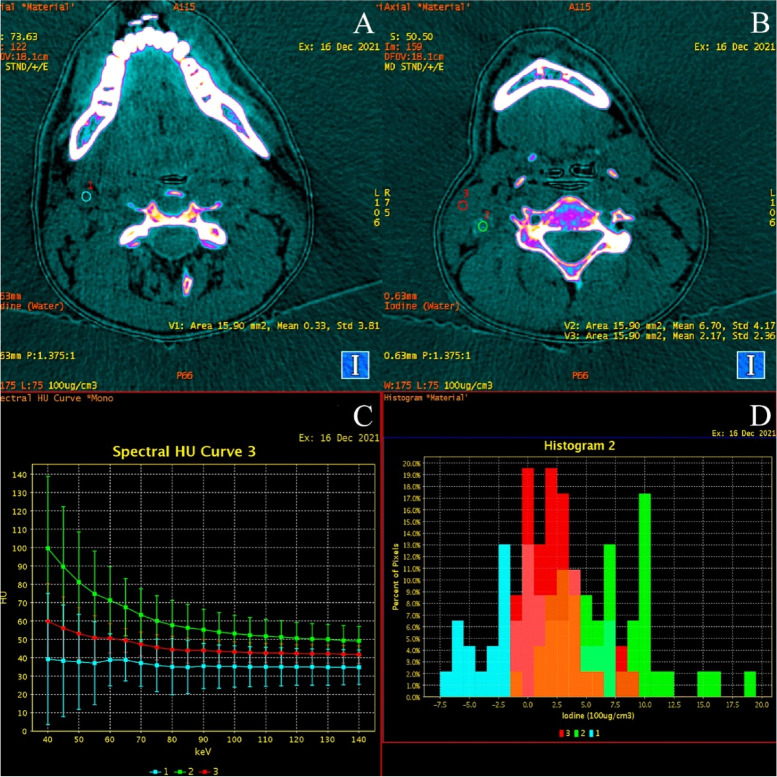
DECT scans of the neck. Suspicious lymph node the same in Fig. 1 (ROI 2 - green), normal lymph node (ROI 1 - blue) and physiological muscle tissue (ROI 3 - red). Analysis with use of color maps depending on the concentration of the endogenous iodine. In pathological tissue, the concentration of accumulated iodine was the highest, it was 6.7 ± 4.17 × 100 µg / cm3, in muscle tissue it was 2.17 ± 2.36 µg / cm3, and the lowest in the normal lymph node 0.33 ± 3.81 × 100 µg / cm3 **A**, **B**. X-ray absorption analysis by pathological tissue (green), muscle (red) and normal lymph node (blue) on graphs showing ionizing radiation absorption curves depending on the photon energy in the range of 40–140 keV in the range of 5 keV based on actual values ​​**C**. Graphical representation of the distribution of dense measurements in ROI 1, ROI 2 and ROI 3 in the form of percentage distributions of pixels, one of the examples of the tumor. All analyses are performed because unique capabilities of the DECT modality

**Fig. 3:**
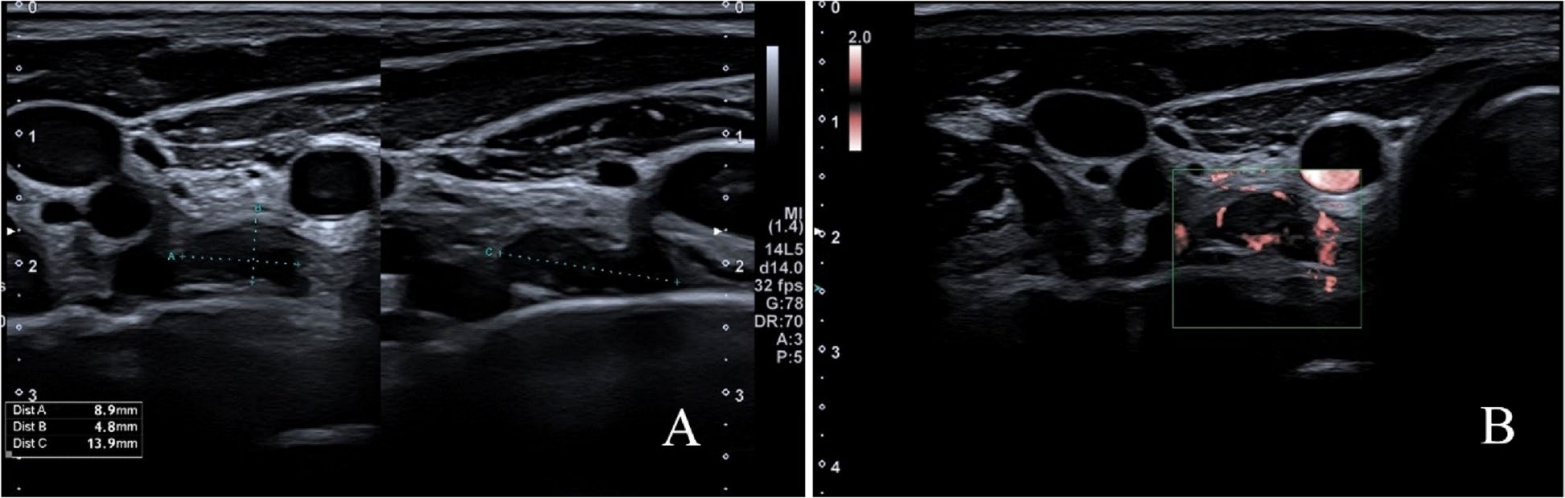
Ultrasound of the neck after thyroidectomy and radioiodine therapy. In postoperative bed presence of previously undetectable mass of hypoechogenic glandular tissue (with increased vascularity) – suspicious of DTC recurrence. (**A**) Transverse/sagittal section (**B**)Transverse section – CDI presentation

**Fig. 4:**
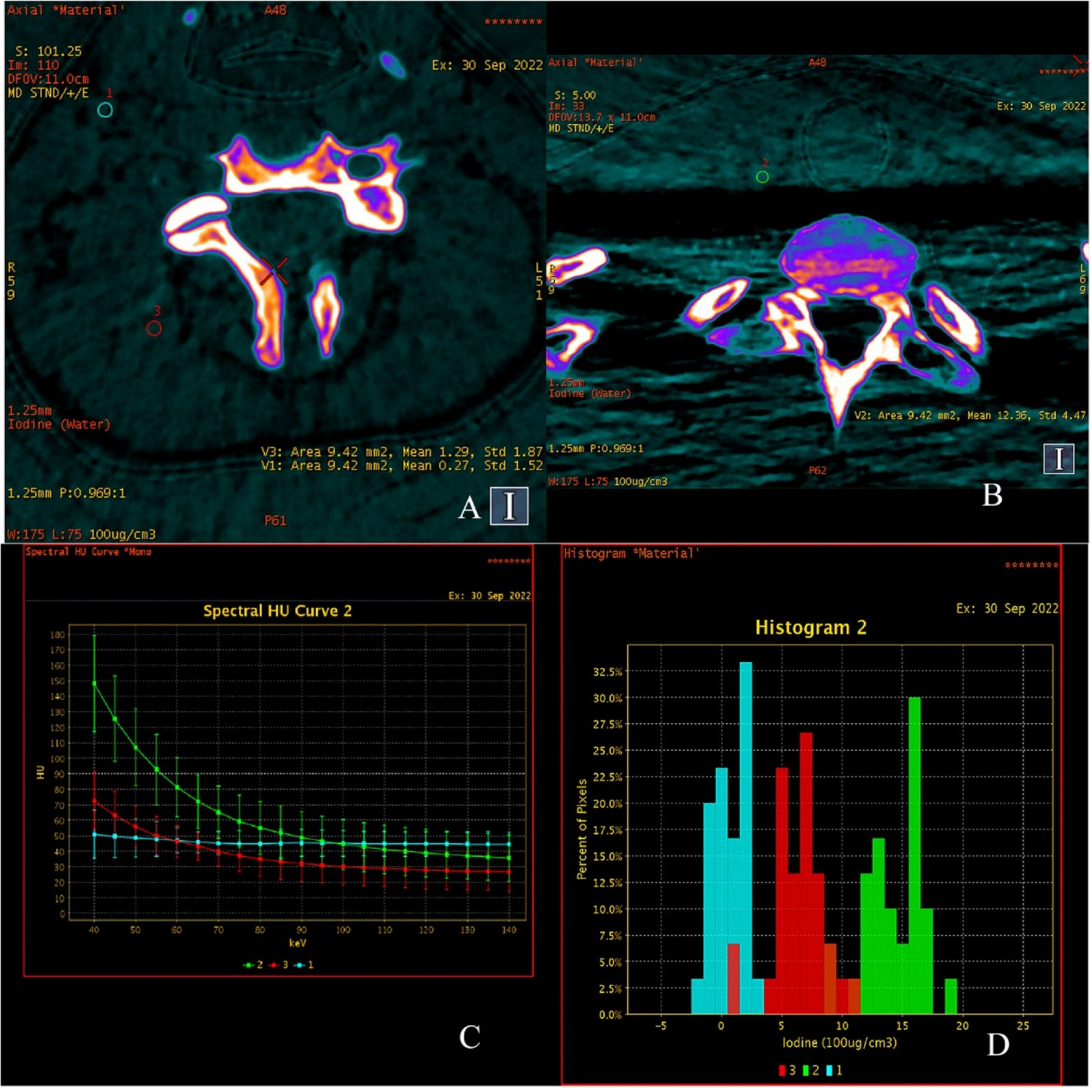
DECT modality analysis of thyroid cancer metastasis to the lymph node. DECT scans show pathological tissue (ROI 2 - green), normal lymph node (ROI 1 - blue) and physiological muscle tissue (ROI 3 - red). Analysis on color maps depending on the concentration of the endogenous iodine. In pathological tissue, the concentration of accumulated iodine was 12.36 ± 4.47 × 100 µg / cm3, in muscle tissue it was 1.29 ± 1.81 µg / cm3, and in the normal lymph node was 0.27 ± 1.52 × 100 µg / cm3 **A**, **B**. X-ray absorption analysis by pathological tissue (green), muscle (red) and normal lymph node (blue) on graphs showing ionizing radiation absorption curves depending on the photon energy in the range of 40–140 keV in the range of 5 keV ​​**C**. Graphical representation of the distribution of dense measurements in ROI 1, ROI 2 and ROI 3 in the form of percentage distributions of pixels, one of the examples of the SPN analysis in the DECT modality

Patient One, a 22-year-old male, was admitted to the hospital for treatment of pT1bN1Mx papillary thyroid carcinoma (PTC). He had found a few lumps located beneath the jaw in midline axis. Ultrasonography (USG) confirmed enlarged lymph nodes (described as reactive lymphadenopathy), and multiply thyroid nodules. Fine needle aspiration was performed giving “*Bethesda V*” result in one lesion of right lobe. After total thyroidectomy with central and lateral lymphadenectomy patient was referred to Department of Endocrinology and Radioisotope Therapy, Military Institute of Medicine – National Research Institute for RAI treatment. During physical examination standard neck USG was performed, showing enlarged lymph node behind right sternocleidomastoid muscle (Fig. 1). Nodule size was 13.6 × 8.5 × 13.7 mm, with increased, mixed vascular image pattern. Because of the young age, lesion features, and lack of primary ultrasound results the decision to perform DECT was made (Figs. 2, 3 and 4). We also performed fine needle aspiration (FNA) for cytology, and thyroglobulin (Tg) concentration which confirmed presence of PTC metastases (Table 1 Part A). In DECT only one focus (the same one found in USG) was qualified for operation. Patient underwent metastasectomy. Biochemical evaluation was made after a month (Table 1 Part B). Output Tg was 0.52 ng/ml [N: 3.5–77], thyroglobulin antibodies (aTg) was 136 IU/ml [N: 0-115 IU/ml] and stimulated Tg (0,9 mg rhTSH administration for two consecutive days) was 5.09 ng/ml. RAI treatment (120mCi) was carried out. Three days after treatment, in total body scintigraphy, two foci of increased accumulation of radioiodine in the center of the neck, and one slight trace on the right side was confirmed. In control 6 months after RAI treatment (Table 1 Part C), output Tg was 0.51 ng/ml, aTg was < 10 IU/ml, and stimulated Tg was 2.38 ng/ml. USG of the neck showed glandular-type-tissue located in postoperative bed on the right side (same side as primary cancer). Another FNA and DECT was performed. FNA was undiagnostic, but DECT confirmed high concentration of endogenous iodine. Total body scintigraphy after 3 days of administration of 3mCi ^131^I showed no pathological accumulation of RAI. Due to USG and DECT results, and undiagnostic result of FNA patient was referred to the another surgery, which confirmed DTC recurrence.

**Table 1:** Diagnostic results in Patient One

	Part A	Part B	Part C
	Initial hospitalization	Results 2 months after metastatectomy	Results 6 months after RAI treatment (120mCi of ^131^I)
USG	Figure 1	-	Figure 3
DECT	Figure 2	-	Figure 4
Tg [N: 3.5–77 ng/ml]	9,77	0,52	0,51
stimulated Tg [N: 3.5–77 ng/ml]	-	5,09	2,38
aTg [N: 0-115 IU/ml]	136	136	< 10
FNAC	High number of thyrocytes with foci nuclei polymorphism, and cytological features of PTC, forming groups and spatial forms Biopsy Tg- 147,8	-	Undiagnostic result

## Case 2

**Fig. 5:**
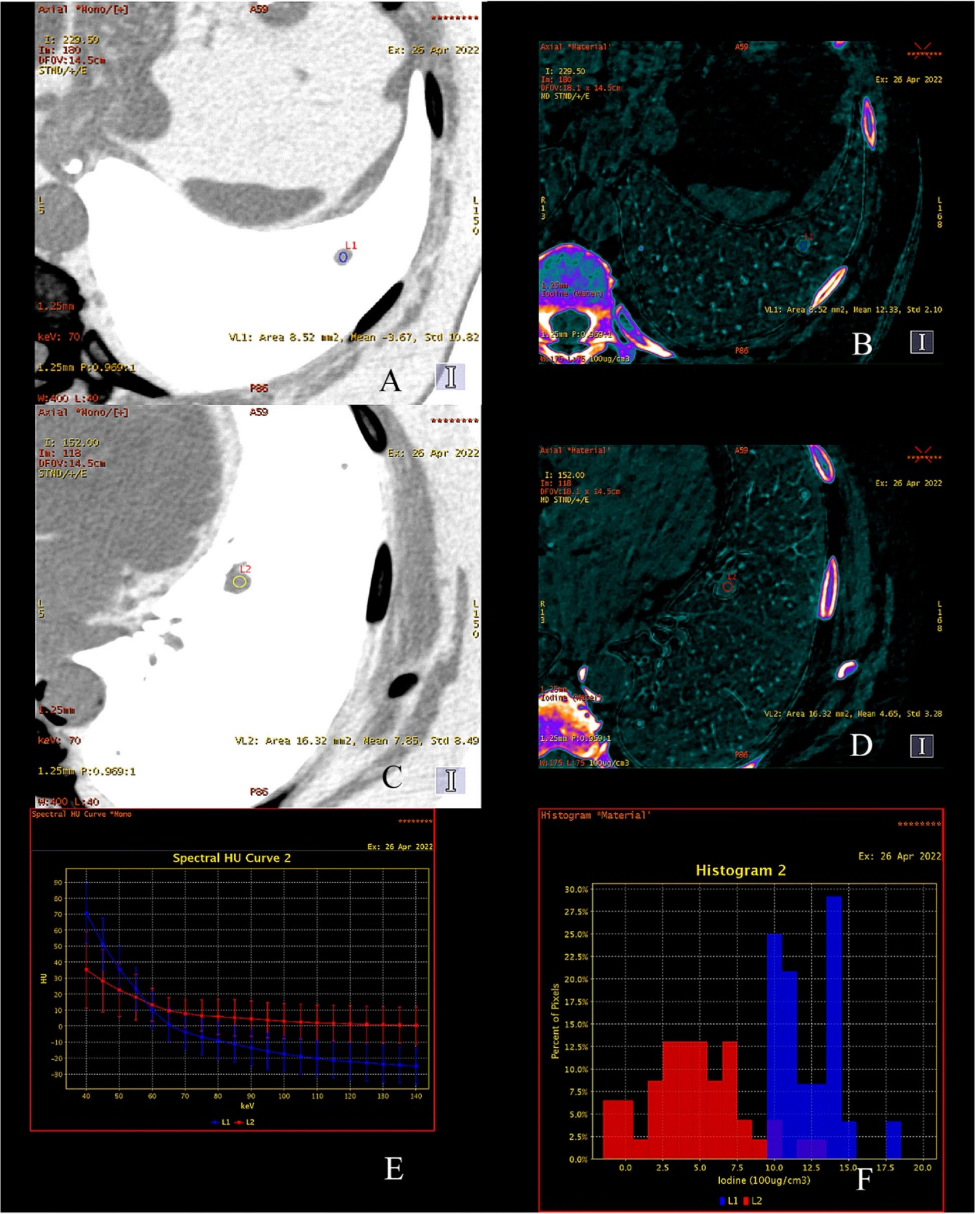
DECT analysis of thyroid cancer metastases to the lung. Solid lung tumor in the left lower lobe of the left lung (L1 - blue). Tumor densities − 3,67 ± 10.82 HU measured on invert gray maps improving visibility of the lesion **A**. High index of endogenous iodine accumulation 12.33 ± 2.10 × 100 µg / cm3 suggest metastasis of thyroid cancer **B**. Analysis of another nodule (L2 - red) also in the lower lobe of the left lung (C, D**)**. Tumor density + 7.85 ± 8.49 HU **C**. Lower index of endogenous iodine accumulation 4.65 ± 3.28 × 100 µg / cm3 **D**. Comparison of X-ray absorption analysis by L1 and L2 in graphs showing ionizing radiation absorption curves as a function of photon energy in the range of 40–140 keV in the range of 5 keV (**E**)

**Fig. 6:**
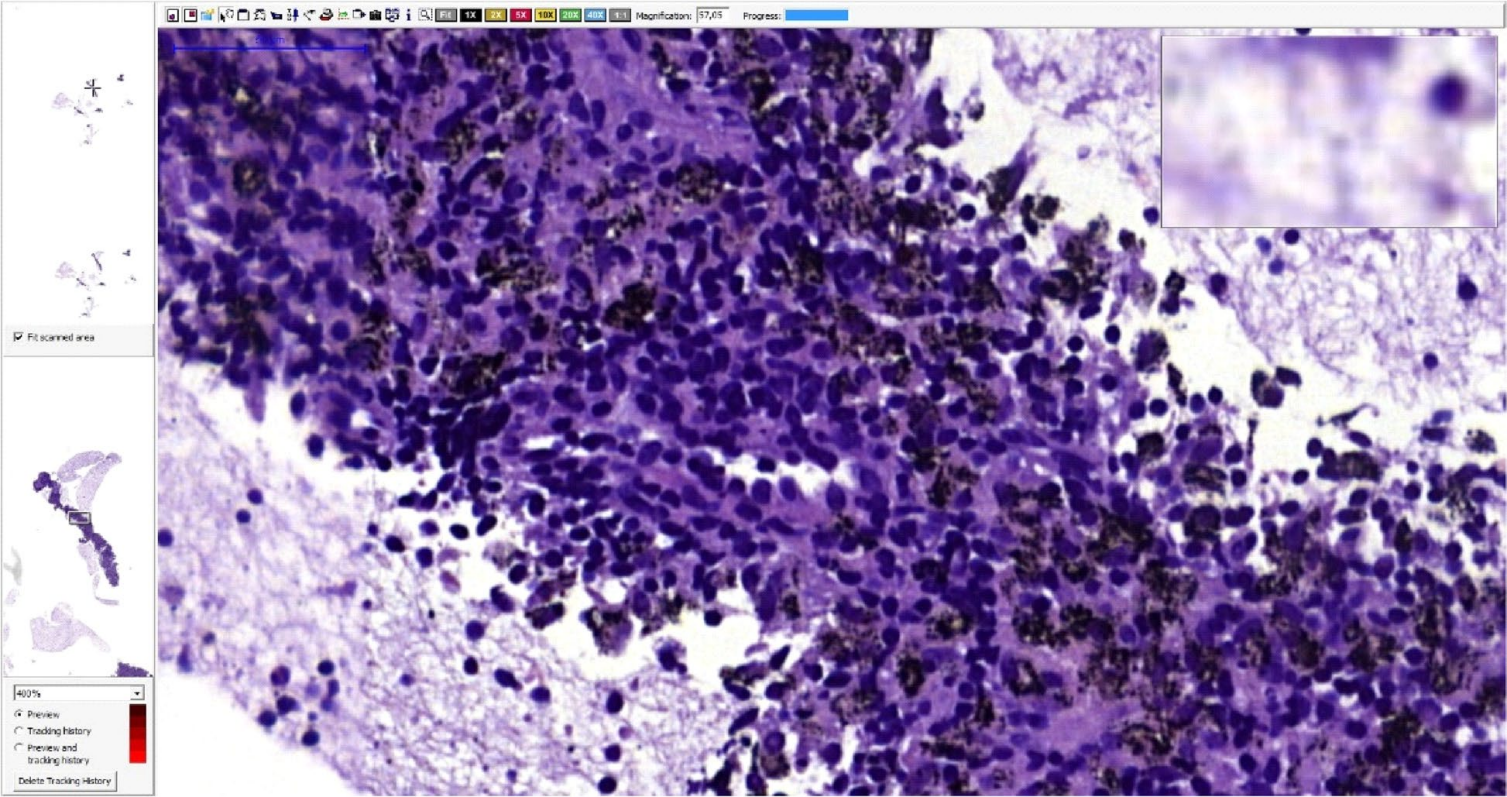
Pneumoconiosis lymphadenopathy. HE staining, magnification – 57x. Black spots represents accumulation of carbon dust deposits

**Fig. 7:**
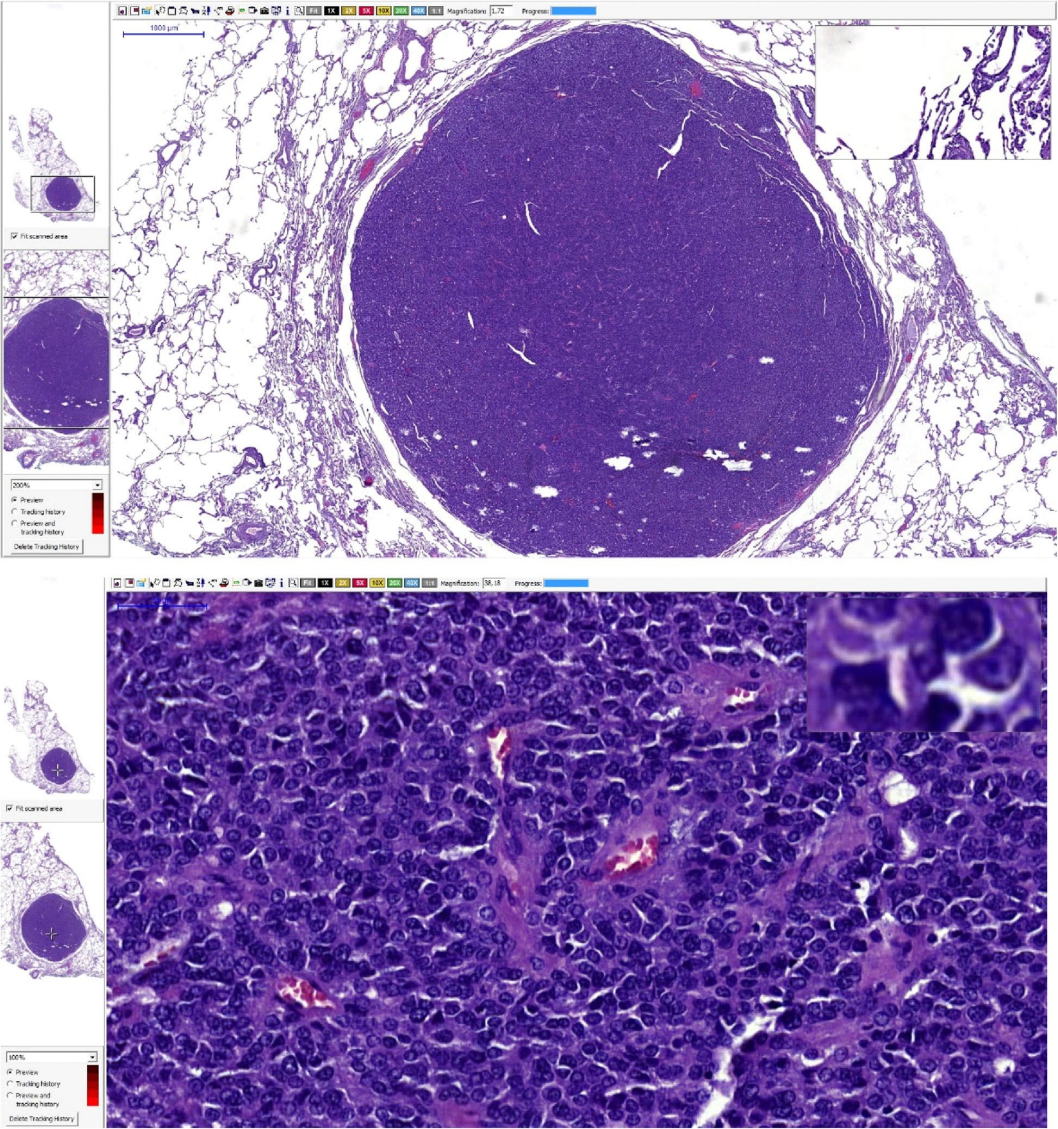
Lung nodule harvested due to DECT locating. HE staining, magnification – 1,72x and 38x

**Fig. 8:**
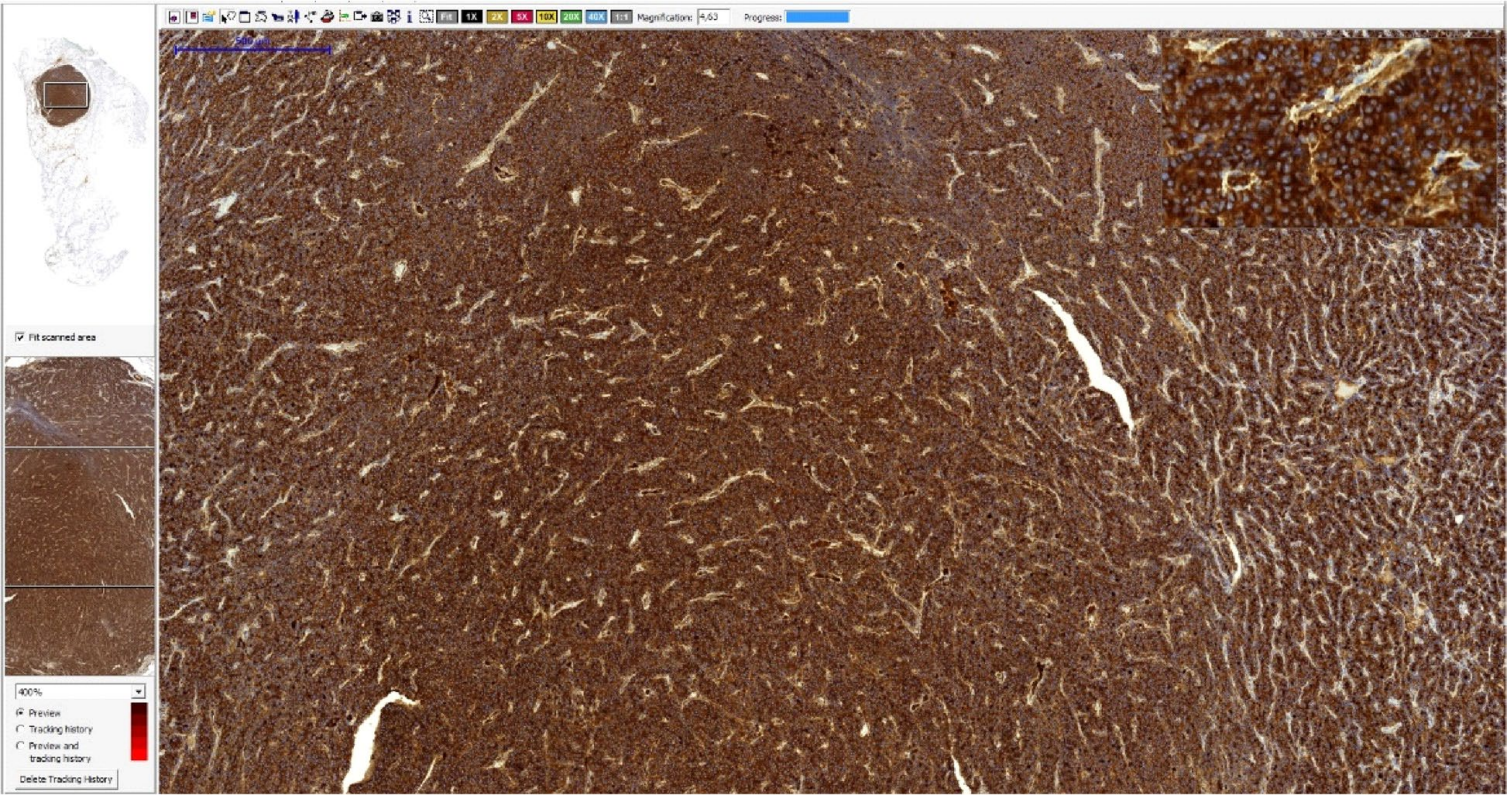
Lung nodule harvested due to DECT locating. Thyroglobulin staining, magnification – 4.63x

**Fig. 9:**
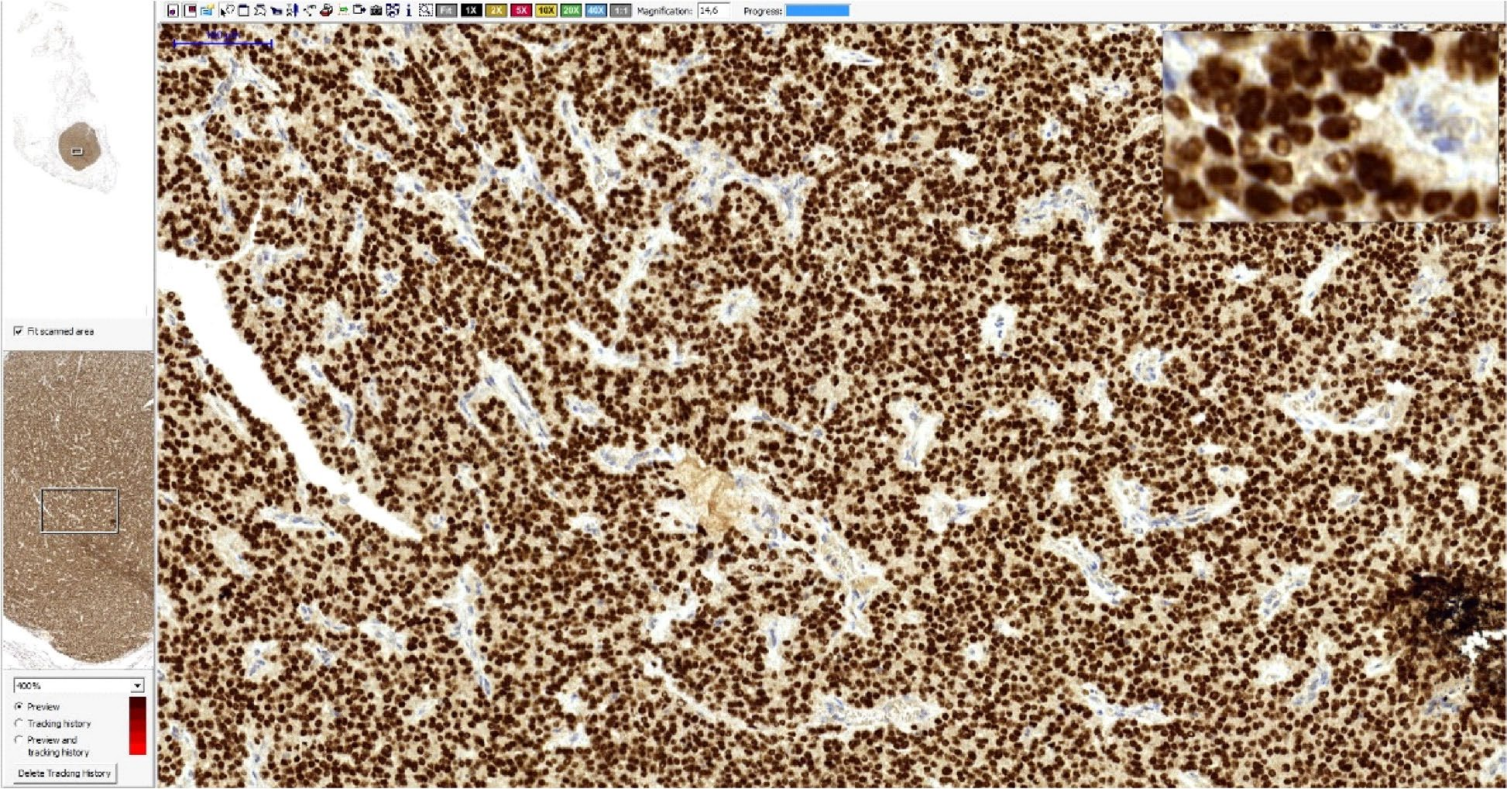
Lung nodule harvested due to DECT locating. TTF1 staining, magnification − 14.6x

Patient Two, a 74-year-old female, was admitted to the hospital for treatment of pT1(m)N0 follicular thyroid carcinoma (FTC). She underwent thyroidectomy with central lymphadenectomy and was referred to the hospital for RAI therapy. During neck USG no sign of residual disease was found. In the day of admission Tg levels was > 500ng/ml [N: 3.5–77] and aTg was 136 IU/ml [N: 0-115 IU/ml]. After rhTSH administration (0,9 mg for two consecutive days) Tg was 28387ng/ml. She received 120mCi of ^131^I. Moreover 18 F-FDG PET was performed. Examination showed metabolically active lymph nodes: subaortic 8 × 6 mm (Standard Uptake Value [SUV] − 4,4), and paratracheal left 8 × 6 mm (SUV − 2,4). There was also morphological presence of multiply pulmonary nodules (up to 12 mm) but with no excessive metabolism of 18 F-FDG. Next, the DECT was performed, and showed high iodine concentration in previously observed lung nodules – giving suspicion of FTC metastasis (Fig. 5). In control 2 months after RAI we noticed output Tg concentration of 24832.00 ng/ml [N: 3.5–77], slightly increased aTg 121 IU/ml [N: 0–115], and stimulated Tg 30767.00 ng/ml [N: 3.5–77]. Patient underwent additional course of RAI therapy (200mCi). Post therapeutic scintigraphy showed no sign of radioiodine accumulation. Suspicious lymph nodes described in PET was biopsied, with a result of pneumoconiosis lymphadenopathy (Fig. 6). Due to divergent results of DECT, biochemical tests, PET, and scintigraphy the decision was made to perform histopathological biopsy of lung. Fragment of the lung with suspicious nodules (typed by DECT) was harvested. Pathological report showed located in the lung parenchyma cancer metastasis with an immunohistochemical profile corresponding to the primary focus in the thyroid gland: Tg(+), TTF-1(+), CDX2 (-), S100(-), EMA(-), ER(-) (Figs. 7, 8 and 9). Patient was qualified to sorafenibe therapy. After 4 weeks of sorafenibe − 200 mg two times a day (BID) - Tg levels decreased to 2020 ng/ml, and aTg titre decreased to 12 IU/ml (both results decreased over 10x). Further increase of sorafenibe daily dose to 400 mg BID decreased Tg even to 1314 ng/ml. Present dose was tolerable, and patient stayed under control of our clinic, with no serious adverse events observed, and stabilization of cancer progression.

Graphical representation of the distribution of iodine accumulation in L-1 and L-2 in the form of percentage distributions of pixels, one of the examples of the metastases **F**. The tumor L1 – blue showed a greater accumulation of endogenous iodine compared to the tumor at the site of L2 – red.

### Commentary and future use of the method

Both cases showed value of DECT in diagnosis of differentiated thyroid cancer. Especially in borderline cases, like in case 2 presented in the text, the method could help to locate DTC metastasis allowing to quick introduce proper treatment.

No use of iodine contrast agent, which can postpone RAI therapy or cause allergic reactions, was the biggest advantage. However, the American Thyroid Association (ATA) guidelines claims that exogenous iodine, like iodine contrast factor, is generally cleared within 4–8 weeks after administration in most patients, and does not postpone RAI treatment [20]. The European Guidelines due to different iodine supply speaks for extending this period to at least 6–12 weeks (the best for 20 weeks), to ensure total iodine clearance [21]. It is also worth noticing that ATA guidelines are based on the study, which was conducted on relatively low (25) number of patients, and included assessment of urine iodine [22]. We have also to remember about potential saturation and accumulation of cells with iodine, which can reduce effectiveness of RAI treatment.

There are no doubts, that primary techniques for diagnosis of DTC metastases are USG and FNAC [20, 21]. However, the guidelines underline the role of contrast-CT as primary examination in locating distant DTC metastases. On the other hand, every CT is increasing radiation of the patient, and iodine contrast can cause allergic reactions, kidney injury or delay of RAI treatment [20].

That is why, we consider non-contrast DECT as an important additional diagnostic tool in patients after thyroidectomy, with biochemical signs of possible metastatic or recurrent disease where neither ultrasound nor RAI scintigraphy, nor 18 F-FDG PET shows localization of DTC metastases. Also patients with contraindications for iodine contrast administration (allergy, kidney disease) can benefit from this kind of examination. Presented cases confirmed that DECT was able to locate cancer metastases, indicate them to surgeon, and after histological confirmation, to introduce suitable therapy.

## Conclusions

Dual Energy Computed Tomography could be a useful method of thyroid cancer diagnosis. It could be also useful when traditional methods (USG, RAI scintigraphy or [^18 ^F]-FDG PET-CT) do not confirm disease, are unavailable or results are ambiguous. Moreover, thanks to non-contrast character of the imaging, it has lower risk, simplicity of performing, and could be offered to the patients with contrast allergy, or kidney diseases. Further studies, on lager group of patients, are necessary to establish the true value of the method.

## Data Availability

Not applicable.
